# Nanoencapsulation of *Ocimum basilicum* L. and *Satureja montana* L. Essential Oil Mixtures: Enhanced Antimicrobial and Antioxidant Activity

**DOI:** 10.3390/antibiotics14020180

**Published:** 2025-02-11

**Authors:** Natalija Đorđević, Kristina Cvetković, Jelena Stanojević, Ivana Karabegović, Dragiša Savić, Dragoljub Cvetković, Bojana Danilović

**Affiliations:** 1 Faculty of Technology, University of Niš, Bulevar Oslobođenja 124, 16000 Leskovac, Serbia; natalija@tf.ni.ac.rs (N.Đ.); kristina.cvetkovic@student.ni.ac.rs (K.C.); jstanojevic@tf.ni.ac.rs (J.S.); savic@tf.ni.ac.rs (D.S.); bojana.danilovic@junis.ni.ac.rs (B.D.); 2 Faculty of Technology Novi Sad, University of Novi Sad, Bulevar Cara Lazara 1, 21000 Novi Sad, Serbia; cveled@uns.ac.rs

**Keywords:** basil, winter savory, essential oil mixture, nanoencapsulation, chitosan-based nanoparticles

## Abstract

**Background/Objectives:** Essential oils (EOs) represent a natural alternative to chemical additives due to their biological activity. This study evaluated the antimicrobial and antioxidant activities of basil and winter savory EO mixtures, their interactions, and the biological potential of chitosan-based nano-encapsulated EO mixtures. **Methods:** Mixtures of basil and winter savory EOs (ratios 1:1, 2:1, 4:1, 8:1, and 16:1) were analyzed for chemical composition via GC–MS. Antimicrobial activity was assessed using minimal inhibition (MIC) and bactericidal (MBC) concentration assays, and interactions were quantified with fractional inhibitory concentration indices (FICIs). Antioxidant activity was evaluated using the DPPH assay, with combination indices used to interpret interaction effects. Chitosan-based nanoparticles were made with the selected oil mixture (2:1), after which characterization and biological activity were performed. **Results:** The EO mixture with 2:1 ratio exhibited the strongest joint activity, with synergistic or additive effect against four out of six analyzed microorganisms. Antioxidant activity improved with higher basil proportions, with the 16:1 ratio achieving the lowest EC50 value of 0.052 mg/mL after 120 min and demonstrating synergistic effects at all tested ratios. Higher basil EO content also masked the strong odor of winter savory EO. The biological activity of chitosan-based nanoparticles was increased by encapsulation of the EO mixture (2:1), with an encapsulation efficiency of 75.39%. **Conclusions:** The EO mixture (2:1) showed best antimicrobial efficacy, with synergistic or additive effects. The nano-encapsulated mixture showed good biological potential with effective and complete odor neutralization. These results indicate the potential of basil and winter savory essential oil mixtures for sustainable food preservation applications.

## 1. Introduction

Essential oils (EOs) are complex mixtures of volatile compounds generated through different biosynthetic pathways in aromatic and medicinal plants [1,2]. These compounds are responsible for various biological properties, with antimicrobial and antioxidant activity as the most significant [1,3]. Among numerous compounds, the most prevalent and most studied EO compounds, due to their antimicrobial activity against various Gram-positive, Gram-negative bacteria strains [4,5,6,7] and molds [8,9,10], are terpenes and terpenoids [11]. In addition to antimicrobial activity, EOs are known for their ability to neutralize free radicals, thus preventing oxidative stress [12]. The antioxidant potential of essential oils is based on the presence of bioactive components, mainly monoterpenes, sesquiterpenes and phenolic acids [13]. Some EOs contain compounds that can indirectly prevent oxidative stress by reducing the ability of agents, like metal ions or singlet oxygen, to trigger oxidative processes [14]. Although the use of EOs as natural alternatives to artificial additives is increasing in the food industry, negative impact of sensory characteristics still limits their wider use. Encapsulation is one of the promising novel tools for overcoming that problem, improving solubility and stability, and providing controlled release of EO bioactive compounds over time. As an innovative technique, it has great potential in providing numerous advantages and addressing the challenges of using free EO in food matrices [15].

Although most EOs possess a broad spectrum of biological properties, many recent studies have confirmed that mixtures of EOs have even better properties, due to the synergistic interactions between their components [16,17]. Synergistic or additive effects are very common in essential oils. Synergism is present when the combined effect of two or more essential oils results in a stronger effect compared to the sum of the effects of individual EOs. Synergistic or additive interaction can contribute to the increased antimicrobial or antioxidant activity of mixtures of different EOs [18]. Among the many EOs, those from the Lamiaceae family stand out due to their complex chemical composition and diverse range of biological activity [12]. Although this family includes around 7000 species, basil (*Ocimum basilicum* L.) and winter savory (*Satureja montana* L.) were the main focus of our research.

Encapsulation is a process applied to improve the activity and protection of bioactive compounds in food products. Through encapsulation, bioactive substances are “trapped” within a polymer matrix [19]. Nano-sized systems provide a larger contact surface and better control over the release of bioactive components compared to larger systems [20]. Nanoencapsulation of essential oils effectively enhances their stability. It prevents degradation, offers protection from external influences, masks the strong smell and taste of essential oils [21], and reduces the loss of volatile components [22,23,24], while increasing the expression of bioactive properties [22,25,26,27]. The process of nanoencapsulation for EOs involves the synthesis, characterization, and application of systems consisting of a biopolymer and an EO. Various types of biopolymers, such as polysaccharides, are most commonly used as carriers for the nanoencapsulation of essential oils [28,29]. Chitosan is a polysaccharide obtained by deacetylating chitin. Its high biodegradability, stability at different pH values, and potential for chemical modification makes it suitable as a carrier in the nanoencapsulation process of essential oils [30,31]. Chitosan-based nanoparticles (CNPs) combine the beneficial properties of chitosan and the encapsulated substance, making them suitable for various applications [32].

Basil is an aromatic plant native to India and South Asia [31]. In basil EO (BEO), linalool, methyl chavicol, methyl cinnamate, and methyl eugenol are reported as dominant compounds [33]. The presence of these components enables BEO to exhibit a range of biological effects. Studies have shown that the antimicrobial activity of BEO is due to its bioactive components, with linalool playing the most important role [34,35,36]. It has been reported that linalool increases the permeability of the microbial cell membrane, facilitating the entry of bioactive components into a cell or resulting in leakage of contents due to cell membrane damage [37]. In addition to its antimicrobial properties, BEO exhibits a high ability to neutralize free radicals and prevent oxidative processes. The antioxidant activity of this oil can be attributed to components such as α-pinene, γ-terpinene, 1,8-cineole, limonene, linalool, and linalyl acetate [38].

Winter savory is a Mediterranean perennial plant that grows in sunny, warm, and rocky areas [39]. The winter savory EO (WSEO) is rich in monoterpenoids, such as thymol and carvacrol [39,40]. The presence of thymol and carvacrol in WSEO contributes to a strong antioxidant effect, while compounds, such as γ-terpinene and p-cymene, play a crucial role in neutralizing free radicals [41,42,43]. The antimicrobial effect of this EO is mainly based on carvacrol’s reaction with the lipids in the microbial cell membrane, disrupting its integrity and permeability [44].

Considering that, in our previous studies [40,45], the antioxidant and antimicrobial activity of basil (*Ocimum basilicum* L.) and winter savory (*Satureja montana* L.) EOs were studied in detail, the aim of the present work is to analyze the antimicrobial and antioxidant activity of five mixtures of BEO and WSEO (1:1, 2:1, 4:1, 8:1 and 16:1 mixing ratios). The purpose of this research is to provide an overview of the type of interaction (synergistic, additive or antagonistic) in order to determine and select the best combination that exhibits synergistic or additive effects on products. Furthermore, encapsulation of the selected EO mixture, as well as the biological potential of such obtained nanoparticles, will be performed to obtain a product with potential use in the food and pharmaceutical industry.

## 2. Results and Discussion

Our previous study confirms the potential of BEO and WSEO as natural antioxidants and antimicrobial agents for the food industry [40,45]. However, the strong odor and unacceptable sensory characteristics of WSEO impose a limit on its use in food matrices. Based on the findings that the components present in EOs, even those present in very low concentrations, due to synergistic or additive effects, can significantly increase biological activity, one of the newer perspectives recommends using mixtures of EOs instead of individual examples [16,17]. Consequently, mixing WSEO with other oils, in our case with BEO, represents an approach that can maintain or improve antimicrobial or antioxidant activity. Combining the pleasant taste and aroma of BEO with the strong antioxidant and antimicrobial activity of WSEO, despite its unfavorable organoleptic characteristics, represents a way to harness the benefits of both EOs even at lower concentrations, while not affecting the organoleptic characteristics of food. In addition, it was important to evaluate the biological activity of BEO and WSEO mixtures in order to assess their suitability for further applications.

### 2.1. Chemical Composition of EO Mixtures

**Figure 1:**
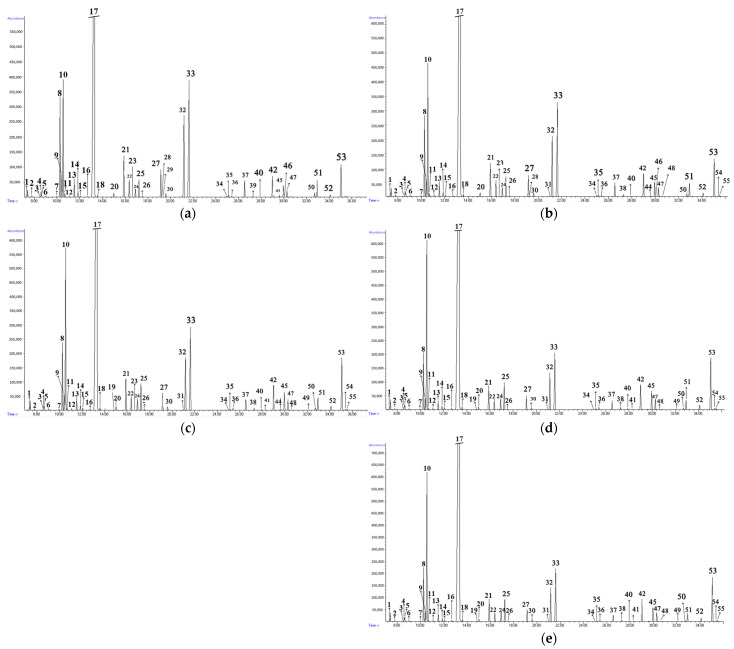
GC chromatograms of *Ocimum basilicum* L. and *Satureja montana* L. essential oil mixtures in ratios: 1:1 (**a**), 2:1 (**b**), 4:1 (**c**), 8:1 (**d**) and 16:1(**e**).

GC–MS analysis showed the presence of a large number of bioactive components and, depending on the mixture ratios, this number could reach 51 (Figure 1, Table 1). GC–MS analysis of BEO and WSEO individually has been analyzed in previous studies [40,45].

BEO obtained by microwave-assisted extraction contained 70 detected compounds, with linalool as the main component at 51.2%, followed by epi-α-cadinol (7.3%) and 1,8-cineole (6.6%) [45]. On the other hand, WSEO obtained by the same extraction process contained 63 detected bioactive compounds, with the highest proportion belonging to carvacrol (18.3%), thymol (12.5%), linalool (10.6%), and p-cymene (9.6%) [40].

GC–MS analysis of mixtures of BEO and WSEO at different mixing ratios allowed the identification of volatile components present in individual EOs [40,45]. According to the significant differences in the chemical composition of the EOs in the mixture, changing the ratio of a particular EO significantly affects the chemical composition. The chemical composition of volatile compounds for all EO mixtures, along with the composition of BEO and WSEO individually, is shown in Table 1. The chromatograms of all EO mixtures (1:1, 2:1, 4:1, 8:1 and 16:1 ratios) are presented in Figure 1.

Considering the content of the main components in individual EOs, changing the EO ratio caused a proportional change in their concentration in EO mixtures. Consequently, domination of linalool is expected in all mixtures, due to its high concentration in BEO. It is worth mentioning that the chemical composition of EO mixtures containing a higher portion of BEO (8:1 and 16:1) are similar, which is explained by the small proportion of WSEO in these mixtures.

Regardless of the BEO and WSEO ratio in mixtures, the most widely represented group of compounds in all mixtures are oxygenated monoterpenes (up to 70.4%). These results highlight the prevalence of oxygenated monoterpenes, such as linalool and 1,8-cineole, in BEO.

The concentration of linalool increases from 42.5% in the 1:1 ratio to 57.3% in the 16:1 ratio. This increase confirmed that linalool is a significant constituent of basil EO, becoming more pronounced as the proportion of this EO increases. Similarly, 1,8-cineo shows a marked increase, ranging from 5.2% at the 1:1 ratio to 7.0% at the 16:1, further emphasizing its predominance in basil EO.

The content of monoterpene hydrocarbons is significantly lower (*p* < 0.05) in the EO mixtures at a higher ratio of BEO, with their concentration ranking from 5.4% in the BEO and WSEO mixture 1:1 to 3.6% in the mixture with the highest ratio of BEO (16:1). This was caused by the dilution of the components characteristic of WSEO as the BEO becomes more dominant in the mixture.

Concentration of sesquiterpene hydrocarbons and oxygenated sesquiterpenes remain almost unchanged in all mixtures, which was expected due to the similar contents of these compounds in both EOs. However, the EO mixture with the lowest BEO content (16:1 ratio) had a significantly lower content of aromatic compounds, which is consistent with the decreasing ratio of WSEO rich in these compounds. Phenylpropanoids content is higher in mixtures with higher content of WSEO in mixture, due to their presence in winter savory EO. Other compounds and trace components remained stable and negligible in all tested ratios.

Among the main identified components, p-cymene, thymol and carvacrol showed a consistently lower concentration in mixtures with lower BEO ratios. This result indicate that these compounds predominantly originate from WSEO, as their relative abundance decreases with increasing BEO content. Limonene showed a relatively stable concentration, varying only slightly between 1.6% and 1.0% in all ratios, indicating its presence in both oils, but in consistently small amounts.

### 2.2. Antioxidant Activity of EO Mixtures

The antioxidant activity of the mixtures of BEO and WSEO at different ratios (1:1, 2:1, 4:1, 8:1 and 16:1) over time (20, 60, and 120 min) are presented in Table 2. The results showed that the antioxidant potential of all mixtures is time-dependent, indicating that the scavenging capacity of the EO mixtures decreases or stabilizes as the reaction progresses.

As previously published, antioxidant activity of individual EOs was in a range from 0.42 to 1.59 mg/mL [40,45]. The literature data confirmed that the antioxidant properties (EC_50_ values) of individual BEO and WSEO, depending on the origins of the plant, chemotype, extraction techniques, and chemical composition ranges from 0.013 to 50 mg/mL [39,40,46] and from 0.032 to 28.9 mg/mL [47,48] for BEO and WSEO, respectively. The significantly higher content of monoterpene hydrocarbons in WSEO, which are known to have exceptional antioxidant activities, is probably responsible for this EO’s better antioxidant activity [49].

The antioxidant activity of the EO mixtures ranged from 0.052 mg/mL to 0.301 mg/mL, taking into account all analyzed time intervals. In the shorter incubation time (interval of 20 min), the degree of neutralization of DPPH radicals was the most pronounced and was statistically significantly different (*p* < 0.05) among all EO mixtures. After 20 min, the 16:1 ratio showed the lowest EC_50_ value (0.078 mg/mL), indicating the highest antioxidant activity. The EO mixture at an 8:1 ratio exhibited similar antioxidant activity with an EC_50_ of 0.099 mg/mL, also reflecting a strong antioxidant effect. In contrast, the mixture with equal content of both EOs (1:1 ratio) showed the weakest antioxidant activity, but was still significantly better compared to both individual EOs. The ratios with intermediate basil proportions (2:1 and 4:1) showed moderate EC_50_ values, of 0.301 mg/mL and 0.257 mg/mL, respectively. These results clearly indicated that higher BEO proportions increase antioxidant activity at this time point. All results obtained at this time interval were statistically significantly different (*p* < 0.05). Similar results were observed at 60 and 120 min. However, the results obtained after 60 min of incubation still indicated a statistically significant difference (*p* < 0.05) in radical scavenging potential between all the mixtures analyzed. Since they showed the highest antioxidant effect, the mixtures at ratios of 8:1 and 16:1 showed statistically significant activity after 120 min of incubation. The remaining mixtures were almost equal in activity (*p* > 0.05).

In general, increasing the proportion of BEO in the mixture increases the overall antioxidant efficacy, which is observed especially in the antioxidant capacities of the EO mixtures at 16:1 and 8:1 ratios. In contrast, WSEO appears to have made smaller contributions to antioxidant activity, as reflected in higher EC_50_ values at lower basil ratios (1:1 and 1:2). On the other hand, although the lowest efficacy was observed for BEO individually, it was considerably improved by mixing with a small amount of WSEO, resulting in maximal antioxidant activity for EO mixtures (16:1 and 8:1 ratios). The exact reason remains unclear, but a possible explanation could be that a high concentration of oxygenated monoterpenes, together with lower concentrations of sesquiterpene hydrocarbons and aromatic compounds from WSEO, contributes more significantly to antioxidant activity compared to mixtures with higher amounts of monoterpene hydrocarbons, aromatic compounds, or other components present in WSEO. Benyoucef et al. [18,49] previously observed that the combination of thymol and p-cymene with chrysanthenone, camphor, and 1,8-cineole results in high antioxidant activity, whereas the absence of thymol and p-cymene significantly reduces this activity.

For each time interval of antioxidant activity determination, the CI index was also calculated in order to define the synergistic, additive or antagonistic effect among the EOs. Since the CI value was less than 1 for all mixtures for all time intervals, the mixtures of BEO and WSEO showed a significant synergistic antioxidant effect. An additive effect was observed for the combination of BEO and fingerroot EO or lemongrass EO [50]. A pronounced synergistic antioxidant effect has been reported in the literature for the combination of various EOs [18,49].

The findings showed that EO mixtures with higher portions of WSEO could be considered as a promising natural antioxidant, capable of significantly decreasing the EC_50_.

### 2.3. Antimicrobial Activity of EO Mixtures

The results of the antimicrobial activity of BEO and WSEO mixtures are presented in Table 3. By comparing the results for individual BEO and WSEO, it is evident that WSEO exhibits significantly higher antimicrobial activity, likely due to the presence and documented antimicrobial activity of thymol and carvacrol, which are more prevalent in this EO [51]. Both EOs and their main components, such as linalool, thymol or carvacrol, are generally recognized as safe (GRAS) and approved for application in food [52] but, nevertheless, the concentration necessary to ensure efficacy, especially for WSEO, could influence sensory acceptance and the overall quality of food. Therefore, by monitoring the antimicrobial activity and antimicrobial interaction of EO mixtures with progressively decreasing WSEO ratios, the main goal was to maintain effectiveness and simultaneously reduce undesirable effects.

As can be seen from Table 3, the minimum inhibitory concentration (MIC) and minimum bactericidal concentration (MBC) values vary depending on the change in the ratio of EOs, but also depend on the type and susceptibility of microorganisms. Regarding activity against *B. subtilis*, the EO mixture at 2:1 ratio showed the lowest MIC (0.20 mg/mL) and MBC (2.05 mg/mL), indicating the highest, and significantly different (*p* < 0.05) antimicrobial activity compared to the other EO mixtures. As the proportion of BEO increased, the MIC and MBC values increased significantly, indicating a decreased efficacy. The lowest activities were recorded in the EO mixture at 16:1 ratio, suggesting that the presence of WSEO was crucial in inhibiting this microorganism.

The lowest MIC (0.23 mg/mL) and MBC (6.46 mg/mL) were recorded for the EO mixture at 1:1 ratio for *S. aureus*, implying a specific interaction between compounds in this mixture that may enhance activity against this gram-positive pathogen. Other EO mixtures showed higher MIC and MBC values, indicating that *S. aureus* is likely to be sensitive to the specific composition of bioactive compounds in the mixtures. The assessment of FICI for this microorganism revealed that an additive interaction was observed just for the EO mixture at 2:1 ratio. This can be explained by the higher content of components with high antimicrobial effect, which mainly originate from WSEO. For other EO mixtures, there were no interaction effects, while an antagonistic effect was observed for the EO mixture at 16:1 ratio. Strong antimicrobial activity against *S. aureus* was previously demonstrated for oregano and thyme EO mixtures, which reduce the MIC value from 6 mg/mL (for the individual oils) to 0.5 mg/mL. This mixture also has a favorable bactericidal effect against *E. coli* and *B. cereus*. The FICI value recorded in this study was between 0.75 and 1, displaying an additive interaction between the two essential oils [53].

The EO mixture at 2:1 ratio also shows the best activity against *P. vulgaris*, with the lowest MIC. As the proportion of BEO increased, the antimicrobial activity decreased. The mixtures with higher content of BEO (1:8 and 1:16 ratios) do not show a statistically significant difference (*p* > 0.05) in activity against this bacterium, indicating that the contribution of bioactive components from winter savory is crucial in inhibiting the growth of this bacteria.

The highest MIC and MBC values for *P. aeruginosa* for all the EO mixture ratios indicate that this bacterium is significantly resistant to these EOs. Almost all mixtures of BEO and WSEO had statistically different effects (*p* < 0.05) on *P. aeruginosa*. However, although the lowest MIC values were obtained for the mixture with equal EOs quantities (1:1 ratio), the FICI values showed that EOs had a synergistic effect on this ratio. Similarly, a 2:1 oil mixture also had a synergistic effect against this bacterium (*p* > 0.05). As the proportion of BEO increases, the antimicrobial activity remains weak, with the weakest effect (MIC 6.53 mg/mL and MBC 26.46 mg/mL) for the mixture with the highest BEO proportion. These results indicate the highest resistance of *P. aeruginosa* to EOs, compared to all other microorganisms tested. In a study by Purkait et al. [54], when analyzing the antimicrobial activity of a combination of black pepper, cinnamon, and clove EOs, *P. aeruginosa* was also found to be the most resistant among the microorganisms tested. The MIC values for the oil mixtures were also lower compared to the individual oils, indicating the existence of an additive or synergistic effect [54].

Regarding activity against *E. coli*, the mixtures at 1:1 and 2:1 ratios showed the best activity, with a significant statistical difference (*p* < 0.05). Higher proportions of BEO led to a decrease in antimicrobial efficacy, indicating a dominant role of WSEO in inhibiting the growth of this bacterium. Previously, it has been confirmed that EOs with predominant contents of thymol and carvacrol (such as oregano oil) can act synergistically with the components of other oils against *E. coli* strains [55]. The same author reported an antagonistic effect between oregano and marjoram essential oils against *B. subtilis* and *S. aureus*, while the same EO mixture showed a pronounced synergistic effect against *E. coli*. The application of mixtures with lower ratios of BEO (1:1 and 2:1) showed a synergistic effect against *E. coli*, while the other mixtures showed no interaction or antagonistic effects (16:1). A synergistic effect (FIC < 0.5) was observed with a 2:1 combination on *K. pneumoniae*. With increasing BEO content, an antagonistic effect among the EOs was observed. Recently, Ayari et al. [56] have shown that BEO combined with eucalyptus, mandarin, oregano, cinnamon, and thyme EOs have additive antimicrobial effects against *B. cereus* [54]. Additive effects against *S. aureus, B. subtilis, E. coli, P. aeruginosa*, and *P. vulgaris* were also observed for different mixtures of clove and rosemary EOs [57].

Generally, the 2:1 combination showed the best results, with synergistic or additive effect against four of six analyzed microorganisms. This result can be explained by the main components of this mixture, including a high content of linalool originating from BEO, along with a high content of thymol and carvacrol derived from WSEO, which exhibit exceptional antimicrobial properties. Carvacrol and thymol have proven antimicrobial effects, which can be explained by their ability to disrupt the cell membrane and create by their high number of bigger pores [17]. Additionally, the presence of p-cimen originating from WSEO induces the swelling of the cytoplasmatic membrane and facilitates the effect of carvacrol and thymol [58]. Linalool, as the main component of the analysed mixtures, exhibits a strong antimicrobial effect by interfering with metabolic pathways in the cells [59]. It has been proven that these components in Eos combinations can evince a synergistic effect, resulting in higher antimicrobial activity [57,60]. As the proportion of BEO increased, antimicrobial activity mostly decreased, indicating the importance of the contribution of the presence of a higher proportion of WSEO. *P. aeruginosa* was the most resistant microorganism, requiring the application of significantly higher concentrations of EO for bactericidal activity. Results indicated that the balance of BEO and WSEO significantly affects antimicrobial activity and that the 2:1 ratio offers the best potential for application for pathogen inhibition in different systems. Given that the EO mixtures with a moderate presence of WSEO also showed better antioxidant activity compared to individual EOs, the EO mixture at a 2:1 ratio was selected for further research and encapsulation.

### 2.4. Characterization of Nanoparticles

The Z-average diameter and polydispersity index of CNPs, BEO:WSEO-CNPs, and BEO:WSEO encapsulation efficiency, along with the results previously published [61] work on CNPs, are shown in Table 4.

For CNPs, the Z-average diameter is 415.4 nm, indicating a larger particle size. In contrast, BEO:WSEO-CNPs have a significantly smaller Z-average diameter of 155.0 nm (*p* < 0.05). This size reduction is likely to be due to the addition of BEO and WSEO, which led to the formation of smaller and more compact NPs. The Z-average diameter measures the average size of NPs in a suspension, which helps to evaluate their size and stability. Smaller particle size (below 200 nm) is considered desirable in various applications, as it increases the surface area to volume ratio and allows better interaction with cellular membranes [62]. In the study of Onyebuchi and Kavaz [63], a similar average particle size of 134.9 nm was recorded for *Ocimum gratissimum* EO-loaded chitosan nanoparticles. The Z-average diameter for *S. montana* encapsulated in a chitosan matrix was 139.9 nm, while the loading of *S. hortensis* EO resulted in particles of 153 nm diameter [64] For *O. basilicum* EO, the particle size ranged from 198.7 nm to 373.4 nm, indicating the existence of smaller and larger particles, which may affect their diffusion. A much larger average size (571 nm) was observed for nanoparticles with *Satureja khuzestanica* EO [65]. Variations in Z-average diameter can arise for several reasons. The technique of nanoparticle synthesis, and process parameters such as mixing speed, temperature, and chitosan concentration, are the main influences on the size of nanoparticles [66]. The pH of the medium can cause insufficient dispersion and the formation of larger particles [67].

The PDI is used to assess the uniformity of the size distribution of nanoparticles, with lower values indicating more homogeneous populations. CNPs showed a PDI of 0.718, indicating a broad size distribution. In contrast, BEO:WSEO-CNPs showed a significantly lower PDI of 0.295 (*p* < 0.05), indicating a more uniform size distribution. Most likely, both for the Z-average and the PDI values, the addition of EOs influenced the stabilization of their values [68]. Previous research has shown that the PDI value for individual *S. montana* L. oil was 0.241 [61]. Another study found that the PDI value for the essential oil of *S. khuzestanica* encapsulated in a chitosan matrix was almost twice as high, at 0.453 [65]. For *O. gratissimum*, the PDI value was 0.288 [63]. The PDI value is mainly influenced by the interaction between the encapsulated active ingredient and the nanoparticle matrix, making it highly complex and difficult to predict in advance [69].

Encapsulation efficiency of 75.39 ± 2.26%, showed that the EO mixtures are effectively loaded into the chitosan. Encapsulation efficiency is the percentage of the active ingredient, i.e., EO, that is successfully encapsulated within a carrier material, such as chitosan nanoparticles [70]. High encapsulation efficiency protects the bioactive compounds and enables their slow, controlled release [71]. There is no available literature data on the efficacy of encapsulation of a mixture of BEO and WSEO. The encapsulation efficiency of BEO directly depends on the concentration of the added EO [70]. Sultan et al. [72] found that the percentage of BEO successfully encapsulated for 2, 1.5, and 1 mL of BEO was 81.96, 65.87, and 48.48%, respectively. Other studies have shown slightly lower encapsulation efficiency of 50.39% for essential oil from *O. basilicum* L. [73] and 61.1% for *O. gratissimum* in a chitosan system [63]. The encapsulation efficiency of members of the genus *Satureja* L. Also depends on the concentration of added EO, as well as the ratio with chitosan, and can range from 46.2% to 97.7% for *S. hortensis* L. EO incapsulated in chitosan matrix [64]. In contrast, a suprisingly low encapsulation efficiency value was recorded for the EO from the same plant (26.57%) [74]. The ratio of added essential oil to chitosan also affected the encapsulation efficiency of other nanoencapsulated oils: peppermint and green tea EOs [75] and *Carum copticum* [76]. Another study showed that the encapsulation efficiency for individual *S. montana* L. EO was 63.2% [61]. In addition to the fact that the efficiency of encapsulation can vary depending on the proportion of essential oil, variations also occur as a result of different properties of essential oils (solubility, polarity), the type and characteristics of the carrier, the encapsulation technique, as well as external conditions (temperature, presence of light, etc.) [77].

#### 2.4.1. Antioxidant Activity of Nanoparticles

The antioxidant activity of CNPs and BEO:WSEO-CNPs are shown in Table 5.

As can be seen, CNPs exhibit EC_50_ values from 46.19 to 39.02 mg/mL for different time intervals, indicating moderate DPPH scavenging activity. On the other hand, with the addition of BEO and WSEO, the antioxidant potential of nanoparticles is significantly improved (*p* < 0.05) due to the presence of the corresponding bioactive compounds. BEO:WSEO-NPs show significantly lower EC_50_ values compared to CNPs, at the same time intervals. Esmaeili and Asgari [76] have shown that there is no significant effect of the addition of *C. captivum* essential oil on antioxidant activity. Compared to chitosan nanoparticles (161 mg/mL), nanoparticles with added peppermint EO had a significantly more pronounced antioxidant effect, with an EC_50_ value of 0.34 mg/mL [75].

#### 2.4.2. Antimicrobial Activity of Nanoparticles

The data presented in Table 6 show the antimicrobial activity of BEO:WSEO-NPs compared to regular CNPs. For all microorganisms tested, BEO:WSEO-NPs consistently showed lower MIC and MBC values, indicating greater potential in inhibiting bacterial growth. For *B. subtilis* and *S. aureus*, BEO:WSEO-NPs showed significantly lower MIC values of 1.58 mg/mL and 1.56 mg/mL, respectively. Similarly, for *P. vulgaris, P. aeruginosa, E. coli* and *K. pneumoniae*, BEO:WSEO-NPs showed lower MIC and MBC values than CNPs, with statistically significant enhancement (*p* < 0.05), especially in bactericidal activity.

The obtained results indicate that the enhanced antimicrobial activity of BEO:WSEO-NPs is due to the presence of bioactive compounds in EOs, which are likely to interfere with microbial activities and cellular processes. Similarly, encapsulated *S. hortensis* successfully reduced the number of *E. coli, L. monocytogenes* and *S. aureus* over a 24-h period [78]. The addition of peppermint EO and green tea improved the antimicrobial activity of chitosan nanoparticles, reducing MBC values by almost 5 times, against *E. coli* and *S. aureus* [76]. Nanoencapsulated BEO demonstrated strong antimicrobial activity against *S. aureus*, and a weaker effect against *P. vulgaris* and *E. coli*, but *P. aeruginosa* and *K. pneumoniae* remained resistant to the effects of these nanoparticles [79].

The antimicrobial potential of essential oils is based on their ability to penetrate the microbial cell membrane, resulting in its depolarization, a disturbance in its permeability, and the leakage of cell contents, such as ions (Ca^2^⁺, Mg^2^⁺, and K⁺), proteins, and nucleic acids [19,80,81,82]. Additionally, coagulation of the cytoplasm may occur, thereby inhibiting enzymes [83]. Nanoencapsulation can prolong the antimicrobial effect of EOs by enabling the slow release of bioactive compounds [84,85]. Due to their small size, nanoparticles can more easily penetrate the microbial cell membrane compared to non-encapsulated oil. This facilitates the direct delivery of the bioactive substances of the encapsulated oil—protected from temperature, moisture, oxygen, and light [86]—into the targeted cells. Once the nanoparticles fully penetrate the cell membrane, their content is released inside the cell, thereby increasing the concentration of the bioactive substance and enhancing its efficacy [87].

## 3. Materials and Methods

### 3.1. EO Mixture Preparation

Raw basil (*Ocimum basilicum* L.) and winter savory (*Satureja montana* L.) were harvested during the flowering period in August 2020 in the area near Leskovac and Rtanj mountain, Serbia. The plant materials were dried for 20 days at room temperature. Essential oils from this plant material have previously been extracted and analyzed [40,45]. Subsequently, microwave-assisted extraction (MAE) was performed using a plant-to-water ratio of 1:10. Specifically, 50 g of dried plant material was mixed with 500 mL of distilled water, and the mixture was heated in the presence of microwaves (800 W) in a microwave oven (Beko, Hertfordshire, UK, MWC 2000 MW) connected to a Clevenger apparatus for 60 min. The essential oils were isolated and separated from the aqueous phase and stored at 4 °C [88]. BEO and WSEO were mixed at five different ratios, 1:1 (5 mL of BEO + 5 mL ol of WSEO), 2:1 (6.66 mL of BEO + 3.33 mL ol of WSEO), 4:1 (8 mL of BEO + 2 mL ol of WSEO), 8:1 (8.89 mL of BEO + 1.11 mL ol of WSEO) and 16:1 (9.41 mL of BEO + 0.59 mL of WSEO). The mixtures were then homogenised by an ultrasonic homogenizer (Witeg, Wertheim, Germany) at 13,500 rpm for 3 min and used for analysis.

### 3.2. Preparation of Chitosan-Based Nanoparticles Loaded with EO Mixture

Chitosan nanoparticles (CNPs) with BEO:WSEO (2:1) mixture were prepared using the ionic gelation method described by Zhang et al. [89]. First, a 0.4% chitosan solution was made by dissolving chitosan powder in 50 mL of 1% acetic acid while stirring with a magnetic stirrer. The solution was filtered to remove insoluble particles, and the pH of the filtrate was adjusted to 4.7–4.8. Tween 60 was then added at a concentration of 0.1%, and the mixture was stirred at 60 °C until a uniform solution was achieved.

Then, 200 µg of a BEO:WSEO (2:1) mixture was dissolved in 4 mL of 96% ethyl alcohol. This solution was added dropwise to the cooled chitosan solution while stirring at 1200 rpm for 30 min. After homogenizing the solution at room temperature, 30 mL of sodium-tripolyphosphate solution (>96% purity, 1.87 mg/mL, pH 4, Centrohem, Stara Pazova, Serbia) was added dropwise, followed by stirring for 60 min. The BEO:WSEO-loaded nanoparticles (BEO:WSEO-CNPs) were obtained by centrifuging the solution at 14,000 rpm for 20 min using an Eppendorf 5418 centrifuge (Eppendorf, Hamburg, Germany). The nanoparticles were stored at +4 °C for future use.

### 3.3. Determination of Encapsulation Efficiency

The encapsulation efficiency was determined by adding 200 µL of BEO:WSEO-CNPs to 5 mL of a 2 mol/L HCl solution. The mixture was incubated in a boiling water bath for 30 min. After cooling, 2 mL of 96% ethanol was added, and the solution was centrifuged at 9000 rpm for 5 min. The absorption spectrum of the resulting supernatant was measured using a 2100 UV spectrophotometer (Cole-Parmer, Vernon Hills, IL, USA) across wavelengths from 200 nm to 400 nm, with a peak absorption wavelength of 275 nm. The weight of EO was determined according to the standard curves and encapsulation efficiency was calculated using the following equation [89]:

(1) $$Encapsulation\;efficiency\left(EE,\%\right)=\frac{weight\;of\;loaded\;BEO:WSEO\;mixture}{weight\;of\;initial\;BEO:WSEO\;mixture}\cdot 100$$

### 3.4. Nanoparticles Characterization

The characterization of CNPs and BEO:WSEO-CNPs, including the Z-average size and polydispersity index (PDI), was carried out using the dynamic light scattering (DLS) technique. The measurements were conducted with the Zeta Sizer 7.11 (MAL1031041, Malvern Instruments Ltd., Malvern, UK).

### 3.5. GC/MS and GC/FID Analyses of EO Mixtures

The qualitative and semi-quantitative analyses of EO combinations were performed on the Agilent Technologies 7890B gas chromatograph coupled with the 5977A mass detector (Agilent Technologies, Santa Clara, CA, USA). The details of the method used are given in Ilić et al. [90]. Semi-quantitative analysis was performed using the external standard method, as explained in Ilić et al. [90], employing standards within the following concentration ranges: β-pinene (0.125–2 mg/mL), 1,8-cineole (0.25–3 mg/mL), limonene (0.5–4 mg/mL), linalool (1.67–15 mg/mL), and γ-terpinene (0.75–5 mg/mL). Each of the thus-obtained concentrations was normalized to obtain a percentage as follows:

(2) $$C_{x}\left(\%\right)=\frac{C_{x}}{\sum C_{x}}\times 100$$

where *C_x_* is the concentration of the individual component in the sample, and Ʃ*C_x_* is the total concentration of all components in the sample.

### 3.6. Determination of Antioxidant Activity of EO Mixtures and Nanoparticles

#### 3.6.1. DPPH Assay

The antioxidant activity of the EO mixtures, CNPs and BEO:WSEO-CNPs were assessed using the DPPH (2,2-diphenyl-1-picrylhydrazyl) assay, based on the method described by Akoto et al. [91], with slight modifications. The samples were dissolved in 96% ethanol, and serial dilutions were prepared at concentrations ranging from 0.5 to 16 mg/mL. A 3 × 10^−4^ mol/L ethanol solution of DPPH (Sigma Aldrich, St. Louis, MO, USA) radicals was added to 2.5 mL of each solution at specific concentrations. The samples were incubated for 20, 60, and 120 min, and their absorbance (As) was measured at 517 nm using a 2100 UV spectrophotometer (Cole-Parmer, Vernon Hills, IL, USA). Absorbance readings were taken for the pure ethanol radical solution as the control (Ac) and for ethanol EO solutions as blanks (Ab). The percentage of DPPH radical scavenging activity was calculated using the following formula:

DPPH radical scavenging (%) = 100 − [(As − Ab) × 100/Ac] (3)

The ability of the sample to neutralize DPPH free radicals was quantified as the EC_50_ value (mg/mL), which represents the sample concentration required to neutralize 50% of radicals during the incubation period.

#### 3.6.2. Evaluation of the Antioxidant Interaction of EOs

To determine the type of antioxidant interactions (synergistic, additive, or antagonistic) between the tested EOs in the mixture, an iso-bologram analysis was conducted based on the median-effect principle (EC_50_). The classical combination index (CI) equation, as described by Purkait et al. [54], was applied to analyze the data. The equation used was:

CI = (D)_1_/(Dx)_1_ + (D)_2_/(Dx)_2_ (4)

where (D)_1_ and (D)_2_ represent the doses (EC_50_ values) of the two EOs in combination, and (Dx)_1_ and (Dx)_2_ are the doses (EC_50_ values) of the individual EOs when tested separately.

The interpretation of the CI values was as follows:

CI < 1: Synergistic interaction (S);

CI = 1: Additive interaction (AD);

CI > 1: Antagonistic interaction (A).

### 3.7. Determination of Antimicrobial Activity of EO Mixtures and Nanoparticles

#### 3.7.1. Determination of Minimal Inhibitory Concentration

The antimicrobial activity of the EO mixtures and nanoparticles (CNPs and BEO:WSEO-CNPs) were evaluated by determining the MIC and MBC values, following the protocol described by Balouiri et al. [92]. The assessment was conducted using the microdilution method against two Gram-positive bacterial strains, *Bacillus subtilis* ATCC 6633 and *Staphylococcus aureus* ATCC 25923, as well as four Gram-negative strains, *Escherichia coli* ATCC 25922, *Proteus vulgaris* ATCC 8427, *Pseudomonas aeruginosa* ATCC 27853, and *Klebsiella pneumoniae* ATCC 700603. The samples were tested at concentrations ranging from 0.6 to 30 mg/mL. Following incubation at 37 °C, results were analyzed using an EZ Read 400 ELISA microplate reader (Biochrom, Cambridge, UK). To confirm the MBC values, samples from EO concentrations that inhibited microbial growth were streaked onto Mueller-Hinton Agar plates (Torlak, Belgrade, Serbia) and incubated at 37 °C for 48 h. The MBC was identified as the lowest EO concentration at which no microbial growth was observed [92].

#### 3.7.2. Assessment of the Interaction of EOs

Combination assays were analyzed using the checkerboard method, as described by Ayari et al. [56]. This approach was employed to determine the fractional inhibitory concentration index (FICI) for mixtures of EOs against different bacterial species. The FIC index was calculated by summing the FICI values of BEO (FICIa), and WSEO (FICIb). These values correspond to the lowest concentrations of each EO and their mixtures inhibiting bacterial growth in the combination tests. The FIC values were determined using the following formulas:

FICIa = MICa (in combination)/MICa (alone)(5)

FICIb = MICb (in combination)/MICb (alone)(6)

FICI = FICIa + FICIb (7)

The FICI value of an EO represents the ratio between the minimum concentration required to inhibit bacterial growth in combination with another EO and the concentration needed to achieve the same effect when used individually. The interpretation of FICI values is as follows:

FICI ≤ 0.5: Synergistic effect (S);

0.5 < FICI ≤ 1: Additive effect (AD);

1 < FICI ≤ 4: No interactive effect (NI);

FICI > 4: Antagonistic effect (A).

### 3.8. Statistical Analysis

All experiments were conducted in triplicate, and the results were expressed as mean values ± standard deviation. Statistical analysis was performed using one-way ANOVA followed by Tukey’s multiple comparison test to identify significant differences among the results. The analysis was carried out using SPSS software version 21.0 (IBM, New York, NY, USA). A *p*-value of less than 0.05 was considered indicative of statistically significant differences between samples.

## 4. Conclusions

The combination of BEO and WSEO enhance biological potential, surpassing the capabilities of individual EOs alone. Although the determination of antioxidant activity showed a synergistic effect among all mixtures, in the case of antimicrobial activity, it is observed that the application of the EO mixture at a 2:1 ratio results in a synergistic or additive interaction between EOs. Characterization of chitosan-based nanoparticles of this EO mixture showed a high encapsulation efficiency and an optimal average nanoparticle size, which ensures good interaction with cell membranes, resulting in good biological activity. Since further research is focused on the application of mixtures of these oils in food preservation systems, nanoencapsulation can contribute to the elimination of the strong aroma, while preserving good antimicrobial and antioxidative potential of this EO mixture.

## Figures and Tables

**Table 1:** Chemical composition of *Ocimum basilicum* L. and *Satureja montana* L. essential oil mixtures at different ratios.

No.	*t*_ret_, Min	Compound	Concentration, %						
			BEO:WSEO Ratio					BEO [31]	WSEO [26]
			1:1	2:1	4:1	8:1	16:1		
**1**	7.32	α-Pinene	0.4 ± 0.00 ^a^	0.4 ± 0.00 ^a^	0.3 ± 0.00 ^a^	0.3 ± 0.00 ^a^	0.3 ± 0.00 ^a^	tr	0.4 ± 0.00 ^a^
**2**	7.78	Camphene	0.3 ± 0.00 ^a^	0.3 ± 0.00 ^a^	0.2 ± 0.00 ^a^	0.2 ± 0.00 ^a^	0.2 ± 0.00 ^a^	-	0.3 ± 0.00 ^a^
**3**	8.51	Sabinene	0.2 ± 0.00 ^a^	0.2 ± 0.01 ^a^	0.2 ± 0.00 ^a^	0.2 ± 0.00 ^a^	0.2 ± 0.01 ^a^	0.2 ± 0.00 ^a^	tr
**4**	8.59	1-Octen-3-ol	0.6 ± 0.00 ^a^	0.5 ± 0.00 ^a^	0.4 ± 0.00 ^b^	0.4 ± 0.00 ^b^	0.4 ± 0.00 ^b^	tr	0.8 ± 0.00 ^c^
**5**	8.65	β-Pinene	0.3 ± 0.02 ^a^	0.4 ± 0.00 ^a^	0.4 ± 0.00 ^a^	0.4 ± 0.00 ^a^	0.4 ± 0.00 ^a^	0.5 ± 0.00 ^a^	-
**6**	9.02	Mircene	0.4 ± 0.00 ^a^	0.3 ± 0.00 ^a^	0.3 ± 0.00 ^a^	0.2 ± 0.00 ^a^	0.3 ± 0.00 ^a^	tr	0.5 ± 0.00 ^b^
**7**	9.96	α-Terpinene	0.3 ± 0.00 ^a^	0.2 ± 0.00 ^a^	0.2 ± 0.00 ^a^	0.2 ± 0.00 ^a^	0.2 ± 0.00 ^a^	-	0.4 ± 0.00 ^a^
**8**	10.26	p-Cymene	6.4 ± 0.09 ^a^	5.0 ± 0.04 ^a^	4.1 ± 0.02 ^b^	3.5 ± 0.00 ^c^	3.7 ± 0.01 ^c^	tr	9.6 ± 0.00 ^d^
**9**	10.41	Limonene	1.6 ± 0.01 ^a^	1.3 ± 0.00 ^a^	1.1 ± 0.00 ^ab^	0.9 ± 0.01 ^b^	1.0 ± 0.00 ^ab^	0.3 ± 0.00 ^c^	2.4 ± 0.00 ^d^
**10**	10.53	1,8-Cineole	5.2 ± 0.12 ^a^	6.0 ± 0.03 ^a^	6.5 ± 0.09 ^ab^	7.1 ± 0.03 ^b^	7.0 ± 0.10 ^b^	6.6 ± 0.00 ^ab^	1.0 ± 0.00 ^c^
**11**	10.68	(Z)-β-Ocimene	0.4 ± 0.01 ^a^	0.3 ± 0.00 ^a^	0.3 ± 0.00 ^a^	0.2 ± 0.01 ^b^	0.2 ± 0.01 ^b^	tr	0.7 ± 0.00 ^c^
**12**	11.07	(E)-β-Ocimene	0.3 ± 0.00 ^ab^	0.2 ± 0.01 ^a^	0.2 ± 0.00 ^a^	0.2 ± 0.00 ^a^	0.2 ± 0.00 ^a^	0.3 ± 0.00 ^ab^	0.4 ± 0.00 ^ab^
**13**	11.52	γ-Terpinene	1.2 ± 0.01 ^a^	0.9 ± 0.00 ^b^	0.8 ± 0.00 ^b^	0.6 ± 0.00 ^c^	0.6 ± 0.00 ^c^	0.1 ± 0.00 ^d^	2.0 ± 0.00 ^e^
**14**	11.85	cis-Sabinene hydrate	0.6 ± 0.00 ^a^	0.5 ± 0.00 ^a^	0.5 ± 0.00 ^a^	0.4 ± 0.00 ^b^	0.4 ± 0.00 ^b^	0.3 ± 0.00 ^c^	1.1 ± 0.06 ^d^
**15**	12.06	cis-Linalool oxide (furanoid)	0.3 ± 0.00 ^a^	0.3 ± 0.00 ^a^	0.3 ± 0.00 ^a^	0.3 ± 0.00 ^a^	0.3 ± 0.00 ^a^	0.2 ± 0.00 ^a^	0.5 ± 0.00 ^b^
**16**	12.68	trans-Linalool oxide (furanoid)	0.3 ± 0.00 ^a^	0.3 ± 0.00 ^a^	0.3 ± 0.00 ^a^	0.3 ± 0.00 ^a^	0.3 ± 0.00 ^a^	0.5 ± 0.00 ^b^	0.3 ± 0.00 ^a^
**17**	13.29	Linalool	42.5 ± 1.06 ^a^	49.1 ± 0.95 ^b^	53.5 ± 1.58 ^c^	57.9 ± 0.62 ^d^	57.3 ± 1.50 ^d^	51.2 ± 1.34 ^bc^	10.6 ± 0.49 ^e^
**18**	13.59	1-Octen-3-il acetate	0.2 ± 0.00 ^a^	0.2 ± 0.00 ^a^	0.2 ± 0.00 ^a^	0.2 ± 0.00 ^a^	0.2 ± 0.00 ^a^	0.2 ± 0.00 ^a^	tr
**19**	14.86	(E)-Ocimene epoxide	-	-	0.3	tr	tr	tr	-
**20**	15.01	Camphor	0.5 ± 0.00 ^a^	0.4 ± 0.00 ^a^	1.9 ± 0.04 ^b^	0.3 ± 0.00 ^c^	0.3 ± 0.00 ^c^	0.3 ± 0.00 ^c^	0.7 ± 0.04 ^d^
**21**	15.90	Borneol	3.0 ± 0.11 ^a^	2.3 ± 0.02 ^a^	0.9 ± 0.00 ^b^	1.6 ± 0.01 ^c^	1.7 ± 0.00 ^c^	1.1 ± 0.04 ^b^	4.8 ± 0.61 ^d^
**22**	16.37	Terpinene-4-ol	1.4 ± 0.00 ^a^	1.1 ± 0.03 ^b^	tr	0.7 ± 0.00 ^c^	0.8 ± 0.01 ^c^	0.4 ± 0.18 ^d^	2.2 ± 0.310 ^e^
**23**	16.65	p-Cymene-8-ol	0.2 ± 0.00 ^a^	0.2 ± 0.00 ^a^	tr	0.7 ± 0.00 ^bc^	0.8 ± 0.00 ^c^	0.6 ± 0.00 ^b^	0.3 ± 0.02 ^a^
**24**	16.92	α-Terpineol	0.7 ± 0.00 ^a^	0.7 ± 0.00 ^a^	0.7 ± 0.00 ^a^	0.8 ± 0.00 ^a^	0.7 ± 0.00 ^a^	1.0 ± 0.05 ^b^	0.6 ± 0.00 ^a^
**25**	17.23	Methyl chavicol	1.5 ± 0.00 ^a^	1.6 ± 0.04 ^a^	1.8 ± 0.00 ^b^	1.8 ± 0.00 ^b^	1.8 ± 0.01 ^b^	2.4 ± 0.15 ^c^	1.1 ± 0.00 ^d^
**26**	17.51	Dihydro-carvone	0.3 ± 0.00 ^a^	0.2 ± 0.00 ^a^	0.2 ± 0.00 ^a^	0.1 ± 0.00 ^b^	0.2 ± 0.00 ^a^	tr	-
**27**	19.15	Carvacrol, methyl ether	2.2 ± 0.17 ^a^	1.7 ± 0.09 ^a^	1.4 ± 0.13 ^b^	1.1 ± 0.02 ^b^	1.1 ± 0.00 ^b^	0.4 ± 0.00 ^c^	4.3 ± 0.04 ^d^
**28**	19.20	Carvone	tr	tr	-	-	-	-	-
**29**	19.41	Thymoquinone	0.2	-	-	-	-	0.4	-
**30**	19.59	Linalyl acetate	0.7 ± 0.00 ^a^	0.6 ± 0.00 ^a^	0.5 ± 0.00 ^a^	0.5 ± 0.00 ^a^	0.5 ± 0.00 ^a^	0.3 ± 0.00 ^b^	-
**31**	20.99	Bornyl acetate	-	0.3 ± 0.00 ^a^	0.2 ± 0.00 ^a^	0.2 ± 0.00 ^a^	0.2 ± 0.00 ^a^	0.3 ± 0.00 ^a^	-
**32**	21.20	Thymol	6.2 ± 0.26 ^a^	4.7 ± 0.12 ^b^	3.8 ± 0.31 ^c^	2.8 ± 0.01 ^c^	2.9 ± 0.00 ^c^	1.2 ± 0.00 ^d^	12.5 ± 0.23 ^e^
**33**	21.65	Carvacrol	9.6 ± 0.47 ^a^	7.4 ± 1.06 ^b^	6.0 ± 0.53 ^c^	4.3 ± 0.82 ^d^	4.6 ± 0.93 ^d^	1.8 ± 0.12 ^e^	18.3 ± 1.36 ^f^
**34**	24.99	Geranyl acetate	0.3 ± 0.00 ^a^	0.2 ± 0.00 ^a^	0.2 ± 0.00 ^a^	0.2 ± 0.00 ^a^	0.2 ± 0.00 ^a^	tr	0.6 ± 0.00 ^b^
**35**	25.14	β-Bourbonene	0.3 ± 0.00 ^a^	0.3 ± 0.00 ^a^	0.2 ± 0.00 ^a^	0.2 ± 0.00 ^a^	0.2 ± 0.00 ^a^	0.3 ± 0.00 ^a^	0.6 ± 0.00 ^b^
**36**	25.41	β-Elemene	0.3 ± 0.00 ^a^	0.4 ± 0.00 ^a^	0.4 ± 0.00 ^a^	0.4 ± 0.00 ^a^	0.4 ± 0.00 ^a^	1.4 ± 0.00 ^b^	0.4 ± 0.00 ^a^
**37**	26.55	(E)-Cariophyllene	1.4 ± 0.02 ^a^	1.2 ± 0.03 ^a^	1.1 ± 0.00 ^b^	1.0 ± 0.00 ^b^	0.9 ± 0.01 ^b^	1.6 ± 0.00 ^c^	2.4 ± 0.03 ^d^
**38**	26.65	α-Guaiene	-	0.3 ± 0.00 ^a^	0.4 ± 0.00 ^ab^	0.4 ± 0.00 ^ab^	0.4 ± 0.00 ^ab^	1.1 ± 0.06 ^c^	0.5 ± 0.00 ^b^
**39**	27.28	α-trans-Bergamotene	0.3	-	-	-	-	-	-
**40**	27.90	α-Humulene	0.2 ± 0.00 ^a^	0.2 ± 0.00 ^a^	0.2 ± 0.00 ^a^	0.2 ± 0.00 ^a^	0.2 ± 0.00 ^a^	0.7 ± 0.02 ^b^	0.2 ± 0.00 ^a^
**41**	28.27	cis-Cadina-1(6),4-diene	-	-	tr	tr	tr	-	0.2
**42**	29.00	Germacrene D	1.8 ± 0.10 ^a^	1.9 ± 0.04 ^a^	2.0 ± 0.09 ^a^	2.0 ± 0.20 ^a^	1.9 ± 0.07 ^a^	5.1 ± 0.06 ^b^	2.5 ± 0.00 ^c^
**43**	29.59	α-Selinene	0.2	-	-	-	-	-	-
**44**	29.96	Bicyclogermacrene	-	tr	tr	-	-	0.3 ± 0.00 ^a^	0.9 ± 0.00 ^b^
**45**	29.96	α-Bulnesene	1.0 ± 0.00 ^a^	1.4 ± 0.01 ^b^	1.4 ± 0.00 ^b^	1.5 ± 0.00 ^b^	1.4 ± 0.00 ^b^	3.4 ± 0.07 ^b^	-
**46**	30.03	β-Bisabolene	0.3 ± 0.00 ^a^	tr	-	-	-	-	1.4 ± 0.00 ^b^
**47**	30.27	γ-Cadinene	0.7 ± 0.00 ^a^	0.8 ± 0.00 ^a^	0.9 ± 0.00 ^a^	0.9 ± 0.00 ^a^	0.9 ± 0.00 ^a^	2.2 ± 0.00 ^b^	0.7 ± 0.00 ^a^
**48**	30.62	δ-Cadinene	-	0.2 ± 0.00 ^a^	0.2 ± 0.00 ^a^	tr	tr	0.3 ± 0.00 ^ab^	0.4 ± 0.03 ^b^
**49**	32.11	(E)-Nerolidol	-	-	tr	tr	tr	0.3	-
**50**	32.71	Spatulenol	0.7 ± 0.00 ^a^	0.5 ± 0.00 ^b^	0.4 ± 0.00 ^b^	0.3 ± 0.00 ^b^	0.3 ± 0.00 ^b^	0.2 ± 0.00 ^c^	1.6 ± 0.06 ^d^
**51**	32.93	Caryophyllene oxide	1.4 ± 0.01 ^a^	1.2 ± 0.00 ^ab^	1.0 ± 0.03 ^b^	0.9 ± 0.00 ^b^	0.9 ± 0.00 ^b^	1.2 ± 0.00 ^ab^	3.0 ± 0.11 ^c^
**52**	34.10	1,10-di epi-Cubebol	0.4 ± 0.00 ^a^	0.4 ± 0.00 ^a^	0.4 ± 0.00 ^a^	0.5 ± 0.00 ^a^	0.5 ± 0.00 ^a^	1.1 ± 0.02 ^b^	0.3 ± 0.00 ^a^
**53**	35.05	epi-α-Cadinol	2.5 ± 0.09 ^a^	2.9 ± 0.20 ^a^	3.1 ± 0.10 ^ab^	3.4 ± 0.40 ^b^	3.3 ± 0.78 ^ab^	7.3 ± 0.32 ^c^	1.8 ± 0.02 ^d^
**54**	35.39	α-Eudesmol	-	0.2 ± 0.00 ^a^	0.2 ± 0.00 ^a^	0.2 ± 0.00 ^a^	0.2 ± 0.00 ^a^	-	0.2
**55**	35.53	α-Kadinol	-	0.2 ± 0.00 ^a^	0.2 ± 0.00 ^a^	0.2 ± 0.00 ^a^	0.2 ± 0.00 ^a^	0.4 ± 0.00 ^b^	-
Total identified (%)			99.8	99.9	99.8	100.0	99.9	97.5	93.5
Compound Groups (%)									
Monoterpene hydrocarbons (**1**–**3**, **5**–**7**, **9**, **11**–**13**)			5.4	4.5	4.0	3.4	3.6	1.4	7.1
Oxygenated monoterpenes (**10**, **14**–**17**, **20**–**22**, **24**, **26**, **28**–**31**, **34**)			56.0	62.0	66.0	70.4	69.9	62.6	22.4
Sesquiterpene hydrocarbons (**35**–**48**)			6.5	6.7	6.8	6.6	6.3	16.4	10.2
Oxygenated sesquiterpenes (**49**–**55**)			5.0	5.4	5.3	5.5	5.4	10.5	6.9
Aromatic compounds (**8**, **23**, **25**, **27**, **32**, **33**)			26.1	20.6	17.1	13.5	14.1	6.4	46.1
Phenyl-propanoides (**25**, **27**, **32**, **33**)			19.5	15.4	13.0	10.0	10.4	5.8	36.2
Other compounds (**4**, **18**, **19**)			0.8	0.7	0.6	0.6	0.6	0.2	0.8

BEO: basil essential oil; WSEO: winter savoy essential oil; *t*_ret_.: Retention time; tr: Component present in trace amounts <0.05%; Different letters indicate statistically different (*p* < 0.05) values in the row.

**Table 2:** Antioxidant activity of *Ocimum basilicum* L. and *Satureja montana* L. essential oil mixtures at different ratios.

BEO:WSEO Ratio	EC_50_ (mg/mL)			CI		
	20 min	60 min	120 min	20 min	60 min	120 min
1:1	0.251 ± 0.003 ^c^	0.218 ± 0.001 ^d^	0.201 ± 0.002 ^c^	0.47	0.70	0.70
2:1	0.301 ± 0.002 ^e^	0.230 ± 0.002 ^e^	0.196 ± 0.003 ^c^	0.55	0.74	0.78
4:1	0.257 ± 0.002 ^d^	0.217 ± 0.002 ^c^	0.201 ± 0.003 ^c^	0.48	0.70	0.80
8:1	0.099 ± 0.002 ^b^	0.079 ± 0.001 ^b^	0.063 ± 0.002 ^b^	0.19	0.25	0.25
16:1	0.078 ± 0.003 ^a^	0.061 ± 0.001 ^a^	0.052 ± 0.002 ^a^	0.14	0.20	0.21
BEO [31]	1.590 ± 0.060 ^f^	0.910 ± 0.020 ^g^	0.630 ± 0.020 ^e^	-	-	-
WSEO [26]	0.800 ± 0.020 ^g^	0.470 ± 0.020 ^f^	0.420 ± 0.020 ^d^	-	-	-

BEO: basil essential oil; WSEO: winter savoy essential oil; CI: combination index. Different letters indicate statistically different (*p* < 0.05) values in the column.

**Table 3:** Antimicrobial activity and antimicrobial interaction of *Ocimum basilicum* L. and *Satureja montana* L. essential oil mixtures at different ratios.

Microorganism		BEO:WSEO Ratio					BEO [31]	WSEO [26]
		1:1	2:1	4:1	8:1	16:1		
*Bacillus subtilis* ATCC 663	MIC (mg/mL)	0.54 ± 0.05 ^b^	0.20 ± 0.09 ^a^	0.57 ± 0.08 ^b^	0.65 ± 0.06 ^b^	1.31 ± 0.07 ^c^	2.91 ± 0.05 ^d^	0.20 ± 0.07 ^a^
	MBC (mg/mL)	2.15 ± 0.08 ^b^	2.05 ± 0.10 ^b^	8.12 ± 0.08 ^e^	4.61 ± 0.09 ^d^	4.56 ± 0.03 ^d^	4.20 ± 0.06 ^c^	0.65 ± 0.28 ^a^
	FICI	2.9 ± 0.14 ^b^	1.0 ± 0.9 ^a^	3.0 ± 0.21 ^b^	3.5 ± 0.18 ^b^	7.0 ± 0.25 ^c^	-	-
	Effect	NI	AD	NI	NI	A	-	-
*Staphylococcus aureus* ATCC 25923	MIC (mg/mL)	0.23 ± 0.09 ^a^	0.31 ± 0.07 ^ab^	0.52 ± 0.05 ^abc^	0.72 ± 0.09 ^c^	1.62 ± 0.02 ^d^	7.76 ± 0.05 ^e^	0.65 ± 0.28 ^bc^
	MBC (mg/mL)	6.46 ± 0.09 ^b^	8.23 ± 0.07 ^d^	8.23 ± 0.13 ^d^	8.98 ± 0.12 ^e^	16.77 ± 0.07 ^f^	7.76 ± 0.12 ^c^	0.81 ± 0.28 ^a^
	FICI	0.4 ± 0.02 ^a^	0.5 ± 0.03 ^a^	0.9 ± 0.09 ^b^	1.2 ± 0.09 ^c^	2.7 ± 0.11 ^d^	-	-
	Effect	S	S	AD	NI	NI	-	-
*Proteus vulgaris* ATCC 8427	MIC (mg/mL)	0.81 ± 0.10 ^b^	0.25 ± 0.05 ^a^	0.49 ± 0.09 ^ab^	0.62 ± 0.01 ^ab^	2.29 ± 0.09 ^c^	5.82 ± 0.34 ^d^	0.32 ± 0.14 ^a^
	MBC (mg/mL)	4.58 ± 0.11 ^bc^	4.23 ± 0.12 ^b^	4.67 ± 0.09 ^c^	5.45 ± 0.08 ^d^	8.46 ± 0.09 ^f^	7.11 ± 0.21 ^e^	0.66 ± 0.28 ^a^
	FICI	2.7 ± 0.20 ^c^	0.8 ± 0.07 ^a^	1.6 ± 0.11 ^b^	2.0 ± 0.16 ^b^	7.5 ± 0.31 ^d^	-	-
	Effect	NI	AD	NI	NI	A	-	-
*Pseudomonas aeruginosa* ATCC 27853	MIC (mg/mL)	0.91 ± 0.03 ^a^	1.35 ± 0.04 ^a^	2.61 ± 0.10 ^b^	4.44 ± 0.07 ^c^	6.53 ± 0.09 ^d^	12.93 ± 0.11 ^e^	2.91 ± 0.37 ^b^
	MBC (mg/mL)	4.61 ± 0.12 ^a^	8.46 ± 0.09 ^b^	16.13 ± 0.11 ^c^	16.13 ± 0.01 ^c^	26.46 ± 0.13 ^d^	15.12 ± 0.14 ^c^	5.82 ± 0.94 ^b^
	FICI	0.4 ± 0.05 ^a^	0.5 ± 0.04 ^a^	1.0 ± 0.09 ^b^	1.8 ± 0.08 ^c^	2.7 ± 0.13 ^d^	-	-
	Effect	S	S	AD	NI	NI	-	-
*Escherichia coli* ATCC 25922	MIC (mg/mL)	0.51 ± 0.09 ^a^	1.03 ± 0.01 ^b^	2.23 ± 0.06 ^c^	3.04 ± 0.14 ^d^	4.43 ± 0.12 ^e^	3.23 ± 0.21 ^d^	0.81 ± 0.28 ^ab^
	MBC (mg/mL)	4.87 ± 0.08 ^c^	4.73 ± 0.12 ^c^	8.43 ± 0.12 ^d^	8.63 ± 0.08 ^d^	8.99 ± 0.05 ^d^	3.88 ± 0.32 ^b^	1.29 ± 0.56 ^a^
	FICI	0.7 ± 0.06 ^a^	1.6 ± 0.12 ^b^	3.4 ± 0.2 ^c^	4.7 ± 0.21 ^d^	6.8 ± 0.29 ^e^	-	-
	Effect	AD	NI	NI	A	A	-	-
*Klebsiella pneumoniae* ATCC 700603	MIC (mg/mL)	0.75 ± 0.03 ^a^	2.29 ± 0.06 ^b^	2.63 ± 0.09 ^b^	3.12 ± 0.11 ^c^	4.19 ± 0.01 ^d^	12.93 ± 0.12 ^e^	0.65 ± 0.28 ^a^
	MBC (mg/mL)	4.76 ± 0.03 ^b^	8.54 ± 0.04 ^cd^	8.34 ± 0.06 ^cd^	8.90 ± 0.07 ^d^	8.07 ± 0.01 ^c^	14.23 ± 0.12 ^e^	1.29 ± 0.56 ^a^
	FICI	1.2 ± 0.11 ^a^	3.7 ± 0.19 ^b^	4.2 ± 0.22 ^b^	5.0 ± 0.15 ^c^	6.7 ± 0.31 ^d^	-	-
	Effect	NI	NI	A	A	A	-	-

BEO: basil essential oil; WSEO: winter savoy essential oil; MIC—minimal inhibitory concentration; MBC—minimal bactericidal concentration. FICI—fractional inhibitory concentration index, on whose value the effect depends: S—synergistic, AD—additive, NI—no interaction, A—antagonistic. Different letters indicate statistically different (*p* < 0.05) values in the row.

**Table 4:** Nanoparticle characterization parameters.

Sample *	Z-Average Diameter (nm)	Polydispersity Index (PDI)
BEO:WSEO-CNPs	155.0 ± 6.3 ^a^	0.295 ^a^
CNPs	415.4 ± 8.0 ^b^ [44]	0.718 ^b^ [44]

* CNPs—chitosan nanoparticles, BEO:WSEO-CNPs—chitosan-based nanoparticles loaded with basil and winter savory essential oils mixture in 2:1 ratio. Different letters indicate significant differences among the samples in the same column.

**Table 5:** Antioxidant activity of CNPs and BEO:WSEO-CNPs.

Sample	EC_50_ (mg/mL)		
	20 min	60 min	120 min
BEO:WSEO-CNPs	24.2 ± 0.01 ^a^	12.02 ± 0.01 ^a^	7.68 ± 0.09 ^a^
CNPs	46.19 ± 0.02 ^b^	41.67 ± 0.06 ^b^	39.02 ± 0.11 ^b^

CNPs—chitosan nanoparticles, BEO:WSEO-CNPs—chitosan-based nanoparticles loaded with basil and winter savory essential oils mixture in 2:1 ratio. Different letters indicate significant difference among the samples in the same column.

**Table 6:** Antimicrobial activity of CNPs and BEO:WSEO-CNPs.

Microorganism		Antimicrobial Activity (mg/mL)	
		Chitosan NPs	BEO:WSEO-NPs
*Bacillus subtilis* ATCC 663	MIC	3.26 ± 0.07 ^b^	1.58 ± 0.04 ^a^
	MBC	6.43 ± 0.06 ^b^	3.36 ± 0.12 ^a^
*Staphylococcus aureus* ATCC 25923	MIC	3.12 ± 0.03 ^b^	1.56 ± 0.17 ^a^
	MBC	6.25 ± 0.09 ^b^	3.25 ± 1.10 ^a^
*Proteus vulgaris* ATCC 8427	MIC	6.30 ± 0.24 ^b^	3.12 ± 0.08 ^a^
	MBC	6.37 ± 0.12 ^a^	6.31 ± 0.08 ^a^
*Pseudomonas* *aeruginosa* ATCC 27853	MIC	12.22 ± 0.08 ^b^	6.25 ± 0.07 ^a^
	MBC	15.43 ± 0.02 ^b^	6.25 ± 0.04 ^a^
*Escherichia coli* ATCC 25922	MIC	1.56 ± 0.05 ^a^	1.49 ± 0.13 ^a^
	MBC	6.28 ± 0.08 ^b^	3.20 ± 0.21 ^a^
*Klebsiella pneumoniae* ATCC 700603	MIC	6.68 ± 0.23 ^b^	3.31 ± 0.12 ^a^
	MBC	12.50 ± 0.19 ^b^	6.18 ± 0.41 ^a^

CNPs—chitosan nanoparticles, BEO:WSEO-CNPs—chitosan-based nanoparticles loaded with basil and winter savory essential oils mixture in 2:1 ratio. Different letters indicate significant difference among the samples in the same row.

## Data Availability

The original contributions presented in this study are included in the article. Further inquiries can be directed to the corresponding author.
